# Cell death in acute lung injury: caspase-regulated apoptosis, pyroptosis, necroptosis, and PANoptosis

**DOI:** 10.3389/fphar.2025.1559659

**Published:** 2025-03-21

**Authors:** Jun Xiao, Lichuan Wang, Bohan Zhang, Ana Hou

**Affiliations:** Department of Pediatrics, Shengjing Hospital of China Medical University, Shenyang, China

**Keywords:** caspase, NLRP3, RIPK, mlkl, inflammation, acute lung injury

## Abstract

There has been abundant research on the variety of programmed cell death pathways. Apoptosis, pyroptosis, and necroptosis under the action of the caspase family are essential for the innate immune response. Caspases are classified into inflammatory caspase-1/4/5/11, apoptotic caspase-3/6/7, and caspase-2/8/9/10. Although necroptosis is not caspase-dependent to transmit cell death signals, it can cross-link with pyroptosis and apoptosis signals under the regulation of caspase-8. An increasing number of studies have reiterated the involvement of the caspase family in acute lung injuries caused by bacterial and viral infections, blood transfusion, and ventilation, which is influenced by noxious stimuli that activate or inhibit caspase engagement pathways, leading to subsequent lung injury. This article reviews the role of caspases implicated in diverse programmed cell death mechanisms in acute lung injury and the status of research on relevant inhibitors against essential target proteins of the described cell death mechanisms. The findings of this review may help in delineating novel therapeutic targets for acute lung injury.

## 1 Introduction

Acute Lung Injury (ALI) and its severe variant, Acute Respiratory Distress Syndrome (ARDS), are prevalent critical illnesses marked by the compromise of the alveolar-capillary barrier, pulmonary edema, and an unregulated inflammatory response, with a fatality rate of 30%–40% (Bos and Ware, 2022; Mokrá, 2020). The etiology is multifaceted, encompassing various causal variables including infection, trauma, and shock; nonetheless, the fundamental pathological mechanism remains ambiguous (Brower et al., 2000; Lentini et al., 2023; Land, 2013).

Recently, the caspase family of proteins has been involved in the regulation of programmed cell death (PCD), including apoptosis, pyroptosis, necroptosis, and PANoptosis (Kesavardhana et al., 2020). PANoptosis has been a central theme in studies exploring ALI processes and treatment approaches, due to its dual role in regulating tissue homeostasis and the immune microenvironment (He et al., 2025). In the context of *Francisella* infection, AIM2-PANoptosis offers immunoprotection (Lee et al., 2021), while ZBP-PANoptosis is a fuel for the cytokine storm observed in COVID-19 patients (Karki et al., 2022).

Traditionally, apoptosis curtails inflammation by caspase-3/7-mediated “silent” death (Fettucciari et al., 2003), while localized and necroptosis depends on hole creation by gasdermin proteins or MLKL-mediated membrane rupture, resulting in the release of many damage-associated molecular patterns (DAMPs) that drive alveolar macrophage overactivation with neutrophil infiltration (Chen et al., 2016). Bronchial epithelial cells, alveolar epithelial cells (AEC), and macrophages express various pattern recognition receptors (PRRs) that effectively identify pathogen-associated molecular patterns (PAMPs) linked to bacterial, viral, or nonspecific infections (Sundaram et al., 2024a). This recognition initiates the synergistic activation of PCD, including necroptosis, pyroptosis, and apoptosis, which collectively contribute to the inflammatory cascade response and disrupt tissue repair processes (Wang et al., 2023). Caspase-8 cleaves GSDMD, facilitating the conversion from apoptosis to pyroptosis, and also regulates necroptosis via the RIPK1-RIPK3-MLKL axis (Schwarzer et al., 2020a). The intersectionality of PCD indicates that inhibiting a single pathway may be inadequate for reversing ALI progression. Targeting core molecules, such as caspase-8 in PANoptosis, or employing combined strategies across death pathways is necessary (Fritsch et al., 2019).

Nonetheless, the precise regulation and functional, focused intervention mechanisms of PCD in ALI remain inadequately clarified. This review will systematically consolidate recent research on the molecular mechanisms of PCD and targeted pharmacological inhibitors, focusing on caspase-regulated cell death. It aims to investigate the dynamic regulation of PCD in ALI and its potential as a therapeutic target, to offer novel insights to overcome the constraints of conventional anti-inflammatory treatment.

## 2 Inflammatory caspases

### 2.1 The structure and function of inflammatory caspases

Caspase can specifically cleave the peptide chain on the aspartate residue of the target protein. The caspase family members are mainly divided into two categories in terms of function (Ai et al., 2024). The first category is inflammatory caspases, including caspase1/4/5/11, which are related to inflammatory cytokine signaling and pyroptosis (de Vasconcelos and Lamkanfi, 2020). In 1989, the discovery of a novel protease sensitive to the activation of pro-IL-1β was documented and named interleukin (IL)-1β converting enzyme (ICE), also known as caspase-1 (Kostura et al., 1989). Caspase-1, the most widely studied member of the caspase family, plays a vital role in processing cytokines, particularly IL-1β and IL-18. In the resting state, caspase exists in the form of inactive pro-caspase-1 and participates in the inflammasome through ASC (Huang et al., 2021). Mouse caspase-11 is a homolog of human caspase-4/5 (Rathinam et al., 2019). Caspase-1/4/11 acquires protease activity through dimerization, with the functional difference between the three lying in the self-processing of the interdomain linker (IDL). The IDL and the CARD domain linker (CDL) between the caspase-1 catalytic subunits undergo autoproteolytic cleavage to produce the active fragment p20/p10, whereas caspase-1 IDL autoprocessing is necessary for cleaving cytokines (Broz et al., 2010). Unlike caspase-1, caspase-11 does not cleave the precursor of IL-1β but can still generate mature cytokines through the N-GSDMD channel. This is mainly because, during GSDMD maturation, other cell-intrinsic signals trigger NLRP3-dependent caspase-1 activation (Kayagaki et al., 2015). Interestingly, the structure and function of human caspase-4 complement its downstream inflammatory targets. First, caspase-4 dimerizes to acquire basal protease activity. Second, caspase-4 D289/D270 IDL site undergoes self-cleavage to generate fully active protease fragments cleave GSDMD to induce pyroptosis, but only the protease fragment generated by autoproteolysis at the D289 site can cleave pro-IL-1β. Importantly, caspase-4/IL-1β signaling is independent of NLRP3 in human macrophages and epithelial cells (Chan et al., 2023). Meanwhile, the structure of pro-IL-18 has an autoinhibitory interaction between the propeptide and the post-cleavage site, but the loss of the propeptide upon caspase-4/5 cleavage facilitates the conformational maturation of IL-18. Caspase-4/5-IL-1β/IL-18 complements the non-inflammasome pathway for pathological inflammatory responses (Shi et al., 2023).

Inflammatory caspases are not only responsible for the processing of inflammatory factors but also for the targeted cleavage of gasdermin protein. Gasdermins are the executors of pyroptosis and are widely expressed throughout the human body with tissue specificity. In humans, the gasdermin family includes DFNB59 and gasdermin A-E (GSDMA-E), the last five of which have gasdermin-N and -C dual domains (Vasudevan et al., 2023). They are implicated in a variety of biological functions and pathological conditions, such as cell proliferation and death, coagulation and thrombosis, and inflammation (Chen and Broz, 2024). GSDMD is the most studied gasdermin, which uses two routes to mediate pyroptosis (Rathinam and Fitzgerald, 2016).

### 2.2 Caspase-1 and inflammasomes

The dominant and canonical mechanism is the NLRP3 inflammasome-dependent pyroptosis pathway, primarily observed in macrophages (Wei et al., 2022). During the innate immune response, inflammasomes serve as responders to intracellular danger signals. Inflammasome sensor molecule is a type of PRR, including Nod-like receptor (NLR) family protein (such as NLRP1/3/12, NLRC4/5) and HIN200 family protein (such as AIM2) (Fu and Wu, 2023; Le et al., 2023).

NLRs are structurally divided into three parts: N-terminal, C-terminal, and middle NACHT. Except for NLRP10, the C-terminals of other NLR family members contain leucine-rich repeat sequences (LRR) (Sundaram et al., 2024a; Chou et al., 2023). NLRs can be divided into different subtypes based on the different domains contained in the N-terminus, namely NLRBs (baculovirus inhibitor of apoptosis protein repeat, BIR), NLRCs (caspase recruitment domain, CARD), NLRPs (pyrin domain, PYD) (Chen et al., 2021).

Pro-caspase-1 is an effector that contains a CARD. ASC (apoptosis-associated speck-like protein containing a CARD) is a bipartite molecule that contains an N-terminal PYD and a C-terminal CARD (Rathinam and Fitzgerald, 2016). ASC recruits pro-caspase-1 through CARD-CARD interactions. In most cases, the assembly of inflammasomes requires the recruitment of ASC through PYD-PYD or CARD-CARD interactions. ASC serves as a bridge protein in the inflammasome to transmit upstream stimulation information to downstream effector proteins.

However, ASC is not required for the assembly of all inflammasomes (Fu et al., 2024). NLRP1 was the first inflammasome discovered that requires ASC to complete its self-assembly. Various viral proteases and Toxoplasma gondii infection can act as NLRP1 activators (Taabazuing et al., 2020). Human NLRP1 relies on its C-terminal CARD to interact with the C-terminal CARD of ASC to recruit ASC. However, mouse homologous NLRP1 (NLRP1a, b, c) can recruit pro-caspase-1 without the presence of ASC. Additionally, the C-terminal domain of NLRP1 has an autoproteolytic domain between LRR and CARD, called FⅡND, and its self-cleavage is necessary for NLRP1 activation (Taabazuing et al., 2020; Gong et al., 2021). Human NLRB consists of only one family member, NAIP. After activation by bacterial proteins such as flagellin, it recruits NLRC4 through NACHT interaction, and the N-terminal CARD adapter protein contained in NLRC4 can directly recruit and activate pro-caspase-1, implying that ASC is unnecessary in this process (Fu et al., 2024; Zhang et al., 2015). NLRC5 is structurally similar to NLRC4, but functionally NLRC5 acts both as an innate immunity sensor and regulates NLRP3 activation and PANoptosome formation (Sundaram et al., 2024b; Zhang et al., 2021).

**FIGURE 1:**
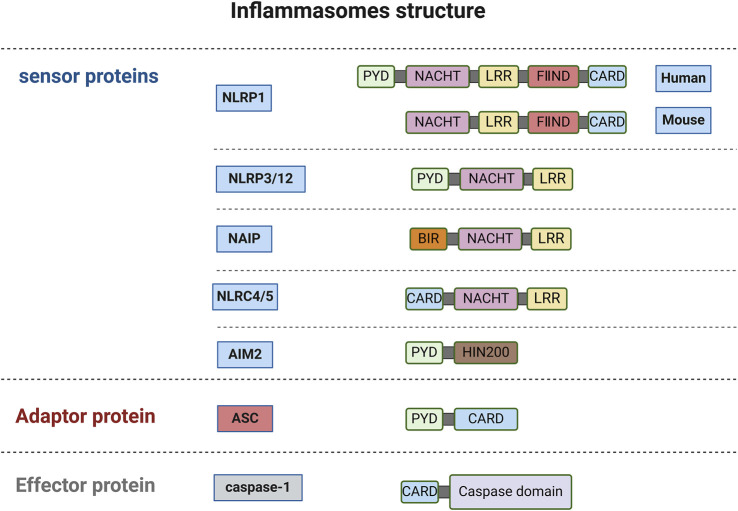
The structure of inflammasomes (created with BioRender.com).

A variety of signals, including entire pathogens, PAMPs/DAMPs, external stimuli that damage lysosomes (e.g., silicon, alum), mitochondrial DNA (mtDNA), and reactive oxygen species (ROS), trigger NLRP3 (Hayward et al., 2018; Yang et al., 2019). Structurally, NLRP3 oligomers transform to monomers or dimers under the regulation of NIMA-related kinase 7 (NEK7); a key process to assemble NLRP3 (Fang et al., 2024). NLRP3 requires expression and assembly signals for activation. PAMPs or DAMPs (such as LPS) first stimulate the TLR/MyD88/NF-κB signaling pathway, which leads to the transcriptional upregulation of NLRP3, ASC, and pro-caspase-1 (Yang et al., 2019). NLRP3 lacks a CARD and recruits ASC through PYD-PYD interactions to form the NLRP3 inflammasome (Fu et al., 2024). Secondly, assembly signals mostly result in K+ efflux that activates NLRP3. Paxillin interacts with NLRP3 and enhances NLRP3 deubiquitination; a process dependent on the ATP release stimulates P2X7R to induce K+ efflux and paxillin phosphorylation, thereby activating the NLRP3 inflammasome (Wang et al., 2020b). The structures and functions of NLRP12 are similar to NLRP3 (Coombs et al., 2024). AIM2 is a cytosolic DNA sensor that recruits pro-caspase-1 through PYD-PYD interactions to promote inflammasome assembly to eliminate harmful pathogenic invasion (Lee et al., 2021) (Figure 1). Consequently, the activated NLRP3 inflammasome activates caspase-1 via ASC signal transduction, cleaving GSDMD, pro-IL-1β, and pro-IL-18 (de Vasconcelos and Lamkanfi, 2020).

### 2.3 Caspase-4/5/11 and the non-canonical pathway of pyroptosis

**FIGURE 2:**
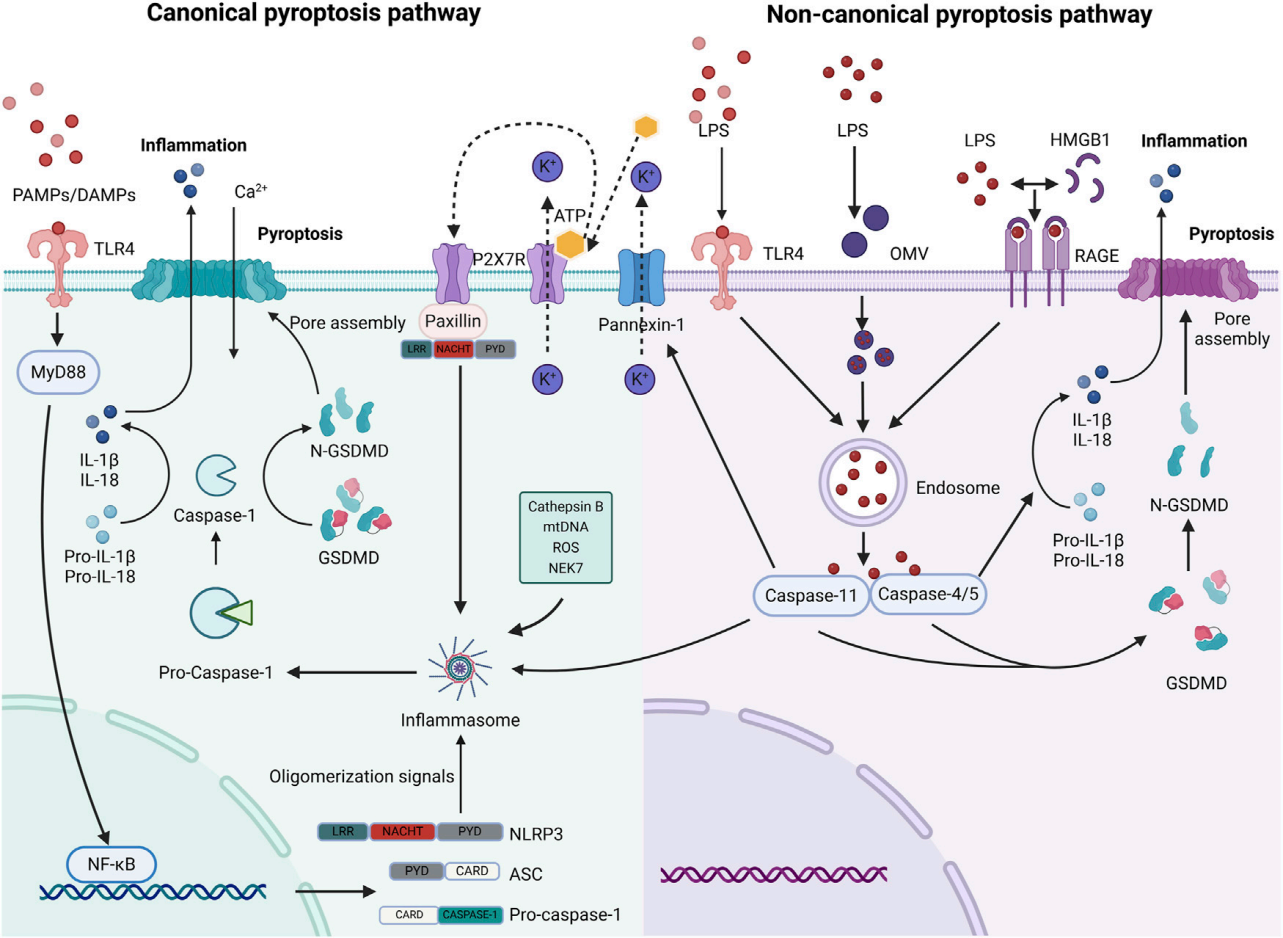
The canonical and non-canonical pyroptosis pathways (created with BioRender.com). The classical process involves the activation of caspase-1 through NLRP3 by PAMPs/DAMPs, K+ efflux, Cathepsin B, mitochondrial DNA (mtDNA), ROS, and NEK7, resulting in GSDMD breakage. In the non-classical process, LPS enters cells through multiple pathways, as illustrated in the diagram. It is promptly identified and triggers caspase-4/5/11 to enzymatically degrade GSDMD. There is interference between the signals on these two paths. The Panniexin-1 receptor mediates the signals, including K+ efflux and ATP release, which in turn stimulates the P2X7 receptor to trigger inflammation via the NLRP3 pathway (Zhang et al., 2023). This could potentially be another intracellular signal that facilitates the transmission of NLRP3 when Caspase-11 triggers pyroptosis.

The non-canonical pathway of pyroptosis mediated by caspase-4/5/11 is different from the canonical pathway mediated by caspase-1. The formation of LPS monomers by extracellular LPS with the help of LPS binding protein (LBP) is the initial step for toll-like receptor 4 (TLR4) to recognize LPS. Subsequently, the LPS monomers-LBP complex is transported to CD14 and combined with TLR4/MD-2 complex to complete the entire process of TLR4 recognition of extracellular LPS (Mazgaeen and Gurung, 2020). In addition, Vishva Dixit et al. demonstrated that caspase-11 could induce pyroptosis by sensing intracellular LPS independent of caspase-1 through an unknown mechanism independent of TLR4. Caspase-11 plays a bigger role than caspase-1 in the lethal inflammatory response (Kayagaki et al., 2011; Kayagaki et al., 2013). LPS can enter the host cytoplasm through a variety of pathways. One mechanism involves LPS binding to high mobility group box 1 protein (HMGB1) and entering cells, which is mediated by the receptor for advanced glycation end products (RAGE) (Matikainen et al., 2020). Contrarily, outer membrane vesicles can serve as carriers for LPS internalization into the cytoplasm (Yi, 2020). Additionally, caspase-4/5/11 directly recognizes cytoplasmic LPS, leading to its oligomerization and activation (Shi et al., 2014). Eventually, caspase-4/5/11 specifically cleaves GSDMD, generating N-GSDMD that disrupts the cell membrane, resulting in the formation of a non-selective transmembrane cavity, the release of cytokines, disruption of water ion balance, and cellular swelling (Wang et al., 2020a) (Figure 2).

## 3 Apoptotic caspases and apoptosis

Apoptosis is a tightly controlled type of programmed cell death that is essential for illness, tissue homeostasis, and biological growth (Kesavardhana et al., 2020). Apoptotic caspases comprise the primary executors of apoptosis, apoptosis-initiating proteins (caspase-2/8/9/10), and apoptosis effector proteins (caspase-3/6/7) (Van Opdenbosch and Lamkanfi, 2019).

Exogenous and endogenous stimuli activate apoptosis via two pathways. First, exogenous stimuli such as pathogens and inflammatory factors are recognized by death receptors, such as Fas and tumor necrosis factor receptor-1 (TNFR-1) (Kim et al., 2024). The ligand activates the death receptor and binds to the Fas-associated death domain (FADD) to recruit caspase-8/10 to form a complex that initiates the downstream caspase chain reaction (Schwarzer et al., 2020a; Jiang et al., 2021). Next, endoplasmic reticulum stress, oxidative stress, cytochrome c, and other non-receptor stimuli induce mitochondrial membrane permeability and pro-apoptotic genes actively participate in this process. Finally, the endogenous and exogenous pathways complete signal transmission, which is regulated by the apoptotic effector caspase-3/7 (Chen et al., 2019).

To guarantee accurate apoptosis, apoptosis regulatory mechanisms include the mitochondrial/death receptor pathway, zymogen activation cascade, repressor protein antagonism, and calpain cross-regulation (Hengartner, 2000). When the death domain, FADD, or apoptotic body (Apaf-1/cytochrome c complex) aggregate, caspase-8/9 is activated (Hengartner, 2000). The starting caspases activate caspase-3/6/7, which are then further cleaved and activated to carry out the apoptotic program (Lakhani et al., 2006). Apoptosis inhibitory proteins, such as XIAP and cIAP, bind directly to the active site of caspase-3/7 to prevent its enzymatic action (Scott et al., 2005). Certain IAPs regulate apoptotic homeostasis by ubiquitinating and degrading the pro-apoptotic proteins Smac/DIABLO (Srinivasula et al., 2001). Bcl-2 and Bcl-xL are anti-apoptotic constituents of the Bcl-2 family. They inhibit the triggering of caspase-9 indirectly by obstructing the release of cytochrome c and the permeability of the mitochondrial membrane (Gottschalk et al., 1996).

Calpains, a category of calcium-dependent cysteine proteases, participate in apoptosis via calcium-dependent activation, direct cleavage of apoptosis-executing proteins, awakening of caspase cascades, and modulation of mitochondrial pathways (Momeni, 2011). In instances of calcium ion overload or endoplasmic reticulum stress, calpains facilitate apoptotic execution through the activation of caspase-3 and caspase-12 by cleavage (Tan et al., 2006). While caspases are the primary executors of pyroptosis, calpains can modulate associated signaling pathways. The activation of calpains may impact the processing of pro-caspase-1 or the construction of inflammasomes, thereby affecting the inflammatory response triggered by pyroptosis (Lopez-Castejon et al., 2012). Furthermore, the GSDMD-induced enhancement of plasma membrane permeability facilitates substantial calcium ion influx into the cell, activating calpain, which subsequently propels various responses linked to pyroptosis, such as intermediate filament disruption, cellular swelling, and rupture (Davis et al., 2019). Nevertheless, additional research is required to clarify their particular role in the focused death signaling network.

## 4 Apoptotic caspases and pyroptosis

Apoptosis and pyroptosis are two separate forms of planned cell death; nevertheless, new research has uncovered intricate interactions between them regarding molecular mechanisms, regulatory networks, and pathogenic processes (Qian et al., 2024). This signaling interference impacts cell fate decisions and is significantly involved in tumor immunity and infection defense. An examination of the architecture and role of inflammatory caspases prompted us to put forward that the primary substrate proteins of caspase-1/4/5/11 are GSDMD, IL-1β, and IL-18. Our investigation into GSDMA-C and GSDME indicated that inflammatory caspase-1, which cleaves GSDMB, can also successfully trigger pyroptosis (Panganiban et al., 2018). We sought to examine whether apoptotic caspases are involved in processing substrate proteins and possess the capability to cleave specific locations, thereby producing active fragments that initiate pyroptosis or other signaling pathways. Caspase-3/8 in apoptotic caspases primarily participates in the pyroptosis pathway by cleaving associated substrate proteins (Jiao et al., 2023; Hou et al., 2020) (Table 1).

**TABLE 1:** Caspase family substrate proteins in pyroptosis.

Inflammatory caspase	Target proteins	Processing sites	Active fragments? (YES or NO)	Ref
Caspase-1	GSDMB GSDMD IL-1β IL-18	D236 D275 D27 D116 D36	YES YES YES YES	Panganiban et al. (2018) Liu et al. (2020a) Exconde et al. (2023) Exconde et al. (2023), Akita et al. (1997)
Caspase-4	GSDMD IL-1β IL-18	D275 D27 D116 D36	YES YES (weakly) YES	Liu et al. (2020a) Exconde et al. (2023) Exconde et al. (2023)
Caspase-5	GSDMD IL-1β IL-18	D275 D27 D36	YES NO YES	Liu et al. (2020a) Exconde et al. (2023) Exconde et al. (2023)
Caspase-11	GSDMD IL-1β	D276 D27	YES NO	Liu et al. (2020a) Exconde et al. (2023)

Apoptotic caspase	Target protein	Processing sites	Active fragments? (YES or NO)	Ref
Caspase-3	GSDMB GSDMD GSDME IL-18	D91 D87 D270 D71 D76	NO NO YES NO	Chao et al. (2017) Chen et al. (2019), Liu et al. (2020a) Kesavardhana et al. (2020), Liu et al. (2020a) Akita et al. (1997)
Caspase-6	GSDMB	D91	NO	Chao et al. (2017)
Caspase-7	GSDMB GSDMD	D91 D87	NO NO	Chao et al. (2017) Taabazuing et al. (2017)
Caspase-8	GSDMC GSDMD IL-1β IL-18	D365 D275 D27 D116 D36	YES YES YES YES	Hou et al. (2020) Kesavardhana et al. (2020), Van Opdenbosch and Lamkanfi (2019), Orning et al. (2018) Exconde et al. (2023), Maelfait et al. (2008) Tummers and Green (2017), Schneider et al. (2017)
Caspase-2/9/10	None	None	None	None

More recent studies have proven that caspase3/GSDME constitutes a new route for cell pyroptosis (Jiao et al., 2023). Caspase-3 cleaves GSDME at the D270 site, resulting in the release of N-GSDME, which oligomerizes and integrates into the cell membrane, causing cell enlargement and rupture (Liu et al., 2020a). When GSDME is substantially expressed in cells, the activation of caspase-3 predominantly induces pyroptosis instead of apoptosis, a mechanism that is especially evident in tissue damage caused by chemotherapeutic agents (Li et al., 2023a; Xing et al., 2023). Viruses operate as activators of GSDME, resulting in pyroptosis in epithelial cells, which serve as the initial line of immunological protection against viruses (Guy et al., 2023). Caspase-8 demonstrates intricate and dynamic pathogenic functions in disease models by regulating cellular pyroptosis, characterized by a “double-edged sword” effect in many illness situations (Liang et al., 2024). Hypoxia or chemotherapeutic drugs like cisplatin can cause caspase-8 to cleave GSDMC or GSDMD, which kills tumor cells or helps blood vessels grow and metastasis spread to other parts of the body through inflammation linked to cell death (Hou et al., 2020; Liang et al., 2024; Xia et al., 2024).

## 5 Caspase-8 and necroptosis

Necroptosis; an alternative pathway of apoptosis, does not form apoptotic bodies and does not require caspase activation for the removal of the damaged cells (Yan et al., 2022). Interestingly, this process can be regulated by caspase-8 (Pang and Vince, 2023). Different stimuli such as TNF, IFN, LPS, DNA, or RNA viruses can trigger necroptosis, which subsequently activates receptor-interacting protein kinase 1 (RIPK1) and RIPK3 and the recruitment of the membrane perforator protein MLKL (mixed lineage kinase domain-like protein) (Yan et al., 2022).

Necroptosis is characterized by a core signaling pathway that includes various protein family members, such as RIPK1/3, the effector protein MLKL, receptor proteins TNFR/ZBP1, and regulatory factors CYLD/cFLIP (Newton and Manning, 2016). The dynamic balance among these components is crucial in determining cell fate. Upon activation of TNFR1 or TLR3/4 and inhibition of the apoptosis key protease caspase-8, RIPK1 interacts with RIPK3 via the RHIM domain to create a necrosome. Subsequently, activated RIPK3 phosphorylates the downstream effector protein MLKL, leading to its oligomerization and insertion into the cell membrane, thereby forming a pore (Alvarez-Diaz et al., 2016). This process compromises membrane integrity, resulting in the release of DAMPs such as HMGB1 and ATP, which in turn initiates a robust inflammatory response (Kao et al., 2014; Tummers and Green, 2017). Furthermore, pathogenic signals like viral RNA can directly activate RIPK3 via ZBP1, circumventing RIPK1 to trigger necroptosis (Yang et al., 2020).

Numerous studies have elucidated the significant role of calpain in necroptosis. Under hypoxia-acidosis conditions, calpain inhibits the caspase-dependent apoptotic pathway by cleaving pro-caspase-3, thereby preventing its activation to active caspase-3 and facilitating necroptosis (Graham et al., 2015). The necroptosis pathway appears to amplify the inflammatory response through the release of DAMPs. In response to cellular DNA damaging agents, the death receptor signaling pathway is activated with calpain’s involvement, resulting in mitochondrial damage through the cleavage of BID (Cabon et al., 2012). Concurrently, mitochondrial damage induces the release of tAIF from the mitochondria into the cytoplasm and nucleus, leading to chromatin lysis and a decline in cell viability (Delavallée et al., 2011). Calpain serves a crucial regulatory role in this process, integrating signals from various pathways and coordinating the initiation and execution of necroptosis (Cheng et al., 2018).

Necroptosis is primarily regulated by ubiquitination modifications, the deubiquitinating enzyme CYLD, and the dynamics of the apoptosis-necrosis homeostasis protein cFLIP (Newton and Manning, 2016). The ubiquitination of RIPK1, facilitated by cIAP1/2 or LUBAC, enhances NF-κB survival signaling and suppresses necroptosis (Bertrand et al., 2008). The deubiquitinating enzyme CYLD facilitates necroptosis through the removal of the ubiquitin chain from RIPK1, thereby enhancing the binding between RIPK1 and RIPK3 (Moquin et al., 2013). cFLIP and caspase-8 possess two death effector domains (DEDs) each, which interact to form a heterodimer via DED-DED binding. cFLP has two isoforms, cFLPL and cFLPS (Irmler et al., 1997). cFLIPL forms a complex with caspase-8 that is inactive, thereby inhibiting the initiation of apoptosis. cFLIPS can entirely inhibit caspase-8 activation, thereby compelling cells to undergo necroptosis (Zhang et al., 2024). Cleavage of CYLD by Caspase-8 may influence its deubiquitinating enzyme activity, thereby regulating the NF-κB signaling pathway, which impacts the apoptotic and necroptotic states of the cell (Jono et al., 2004). RIPK1/3 serves as a substrate for caspase-8 cleavage. While the direct cleavage of RIPK1/3 by caspase-8 has not been extensively studied, it is evident that this process can reduce inflammation and modulate the interplay between apoptosis and necroptosis (Schwarzer et al., 2020b).

Necroptosis serves a dual function in the context of infection defense and inflammatory diseases, facilitating the elimination of abnormal cells while also potentially intensifying tissue damage. Host detection of viral nucleic acids through ZBP1 initiates MLKL-mediated cell membrane rupture, resulting in the release of viral particles and the activation of an antiviral interferon response (Karki et al., 2022; Li et al., 2023b). Excessive necroptosis results in the release of significant quantities of IL-1α and IL-33 (Li et al., 2020; Karki et al., 2021), which subsequently activate neutrophils and macrophages, thereby contributing to irreversible inflammation in critically ill COVID-19 patients (Li et al., 2020).

## 6 Caspases and PANoptosis

Recent studies have found that the immune system releases several PAMPs and DAMPs in response to pathogenic infection, which are recognized by numerous inflammasome sensors and cause a variety of programmed cell deaths. Since 1989, when caspase-1 was first characterized as a cysteine-aspartate protease, the caspase family has become integral to the study of programmed cell death (Kostura et al., 1989). Their functions have evolved from the original role in apoptosis to encompass the regulation of new forms of cell death, including pyroptosis and necroptosis, thereby establishing a multi-pathway intersecting PANoptosis network (Qi et al., 2023). The caspase family is crucial in regulating and executing PANoptosis, with caspase-1/8 involved in the initiation phase and caspase-3/7 in the execution phase (Pandeya and Kanneganti, 2024).

Caspase-1 cleaves GSDMD, resulting in pore formation in the cell membrane and subsequent cell lysis (Sundaram et al., 2024a). The membrane rupture observed is indicative of pyroptosis, thereby contributing to the lytic characteristics of PANoptosis (Han et al., 2023). Caspase-8, previously considered an apoptosis promoter, is now recognized for its role in activating the pyroptosis pathway through the cleavage of GSDMC/GSDMD (Hou et al., 2020, Xia et al., 2024), similar to the function of caspase-1. The assembly of PANoptosomes facilitates the interaction of caspase-8 with RIPK1 and RIPK3 (Pandeya and Kanneganti, 2024), which are critical molecules in necroptosis, thereby linking the signaling pathways of apoptosis and necroptosis (Schwarzer et al., 2020b).

Caspase-3/7, primarily responsible for executing apoptosis, is also activated during PANoptosis and works in conjunction with caspase-8 to initiate the apoptotic process. This activation results in the cleavage and activation of downstream substrates that facilitate apoptosis-related changes in cellular morphology (Qi et al., 2023; Hengartner, 2000).

Under immunofluorescence visualization, ASC, caspase-8, and RIPK3 are essential components (Wang et al., 2022). Due to differences in other components, PANoptosome is classified into six classes: TNF-α and IFN-γ-, Z-DNA-binding protein 1 (ZBP1-), RIPK1-, NLRC4-, NLRC5-NLRP12-, and AIM2- (Pandeya and Kanneganti, 2024; Sundaram et al., 2024b; Sundaram et al., 2022).

**FIGURE 3:**
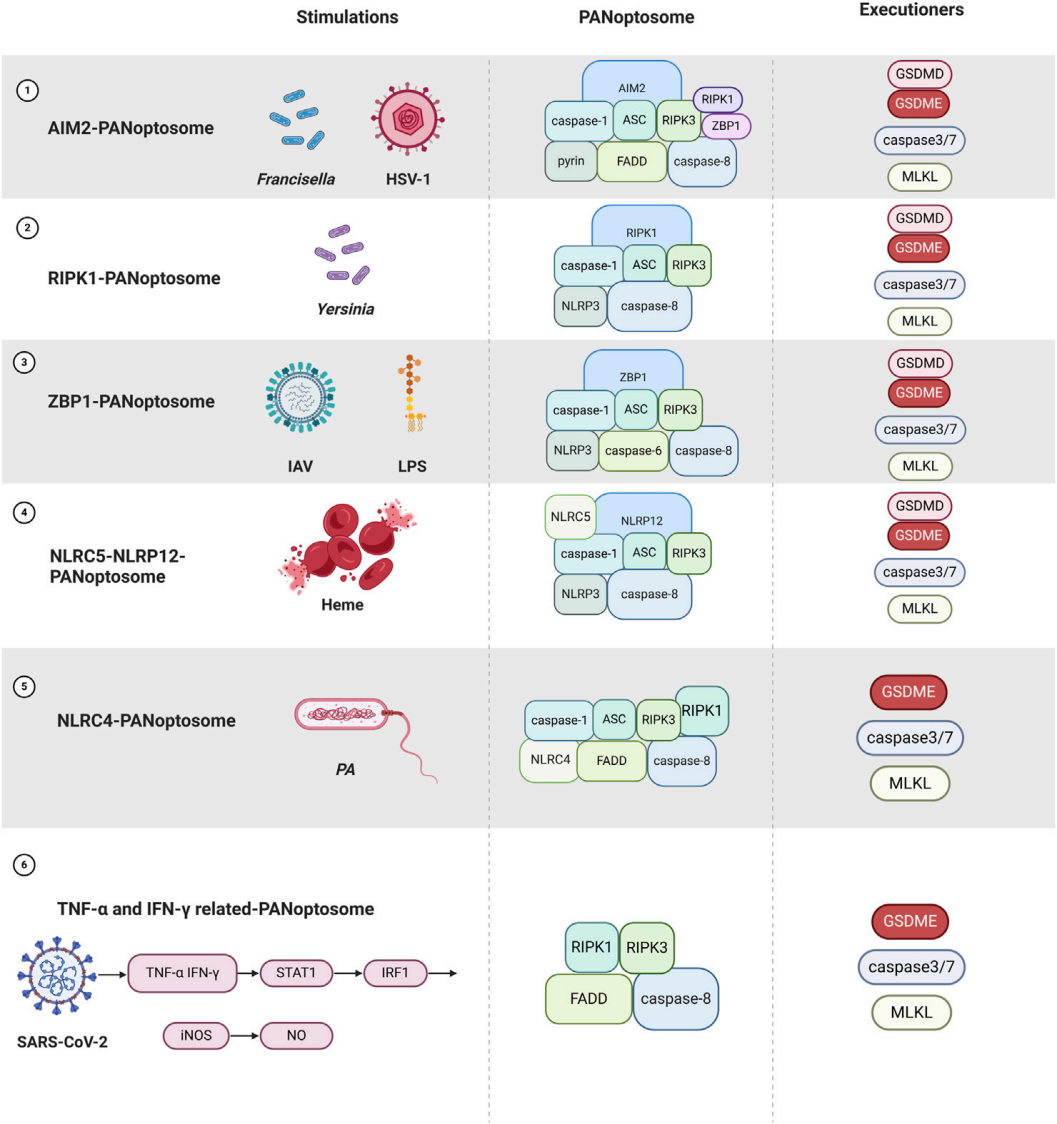
Overview of different PANoptosome assemblies in response to different stimuli as well as PANoptosis occurred (Created with BioRender.com).

The regulation of PANoptosis is intricate and relies on dynamic interactions within the PANoptosome (He et al., 2025). Upstream sensors, including ZBP1 and AIM2, trigger PANoptosome assembly in response to diverse inflammatory signals, such as PAMPs and DAMPs. ZBP1, upon stimulation, recruits caspase-8 and RIPK3 to assemble the PANoptosome (Lee et al., 2021; Zheng and Kanneganti, 2020). PANoptosome forms the regulatory cell death molecules GSDME/GSDMD, caspase-3/7, and RIPK3/MLKL, leading to PANoptosis (Qi et al., 2023) (Figure 3).

## 7 Caspases and ALI

### 7.1 Bacteria-related ALI

Bacteria invade the lungs causing acute infections in adults or neonates when the body is immunocompromised. Especially in neonates with imperfect immune function, microbial infections bring adverse outcomes. Primarily due to the evolution of multiple mechanisms for pathogens to evade host immune surveillance, inadequate or delayed pathogen clearance leads to pathogen proliferation and an overactive immune response inducing ALI (Blutt et al., 2023).

#### 7.1.1 Group B streptococcal (GBS)

GBS is the main causative agent of intrauterine infections and preterm labor as well as neonatal pneumonia and sepsis and is rich in virulence factors (cell wall surface β-protein, capsular polysaccharide, β-hemolysin/cytolysin, hyaluronidase, and membrane vesicles), which help GBS to evade the recognition of the immune system and are involved in cell death and promotion of inflammatory cell recruitment (Furuta et al., 2022).

GBS immune escape and recognition is the first step in GBS lung invasion. The terminal sialic acid of capsular polysaccharide, as well as the β-protein, can be recognized by sialic acid-binding immunoglobulin-like lectin expressed by different immune cells and can inhibit phagocytosis by immune cells (Carlin et al., 2009; Fong et al., 2018). Apoptosis, a non-inflammatory cell death, favors pathogen survival in large logarithms. β-hemolysin induces macrophage apoptosis in a caspase-independent and caspase-dependent manner, and a reduction in the number of macrophages attenuates or delays the onset of specific immune responses (Fettucciari et al., 2003; Fettucciari et al., 2000). In addition, β-hemolysin-induced cytotoxicity is present in invasively infected lung interstitial cells, a process dependent on apoptosis-associated caspases to drive infection progression (Kling et al., 2013). Hyaluronic acid (HA) is a normal component of lung tissue and facilitates interstitial development. In a variety of lung diseases, such as idiopathic pulmonary fibrosis, higher lung HA content is suggestive of disease progression and severity (Juul et al., 1996). Interestingly, GBS specifically secretes hyaluronidase to break down HA and reduce lung HA content, an unconventional phenomenon that seems to favor lung parenchymal invasion (Coleman et al., 2021). Membrane vesicles are pathogenic structures produced by GBS that carry virulence factors and are classified into hemolytic and common membrane vesicles (Surve et al., 2016; Armistead et al., 2021). Hemolytic membrane vesicles carry mainly β-hemolysin/cytolysin, which is associated with high mortality in neonatal mice, and activation of macrophage pyroptosis signaling during fetal life is detrimental to fetal survival (Armistead et al., 2021; Whidbey et al., 2015). Pore-forming toxins of pathogenic bacteria induce multiple forms of cell death and alter normal cellular function to promote pathogen survival *in vivo* (González-Juarbe et al., 2015). For example, necroptosis occurs in macrophages during *Staphylococcus aureus* and *Serratia marcescens* infections (Kitur et al., 2015). Although it has been shown that GBS common membrane vesicles carry this virulence factor, there appears to be no definitive study indicating whether GBS pore-forming toxins cause necroptosis (Surve et al., 2016).

Pyroptosis dominates the immune defense process. Neutrophils are the main source of IL-1β production in lungs infected with GBS, which is directly recognized and facilitates the amplification of inflammatory signals through the TLR (Mohammadi et al., 2016). Macrophages also bear a part of IL-1β production, acting in a sialic acid-dependent or non-dependent manner to enhance NLRP3 activation (Tsai et al., 2020). Apoptosis, pyroptosis, and necroptosis appear to have their roles during GBS infection, but it should be emphasized that erythrocytes are substrates for β-hemolysin and have a high affinity for cell membrane phospholipids (Whidbey et al., 2015; Rosa-Fraile et al., 2014). Heme released from damaged erythrocytes is a threat to the innate immune system, and NLRP12 is an innate inflammasome sensor for heme and PAMPs in bone marrow-derived macrophages (Henkel and O'Neill, 2023). NLRP12 interacts with NLRP3, ASC, caspase-1, caspase-8, and RIPK3 to form NLRP12-PANptosome complexes in response to heme and PAMPs in the hemolysis model induced by heme and PAMPs (Sundaram et al., 2023; Bernard and Broz, 2023). Furthermore, NLRC5 is also involved in interactions driving inflammatory cell death (Sundaram et al., 2024b).

#### 7.1.2 *Yersinia pestis*

Flagellin secreted by the functional type III secretion system (T3SS) of extracellular opportunistic pathogenic bacteria is required for NLRC4 activation (Schubert et al., 2020). Macrophage NLRC4 activation reduces bacterial clearance in an ALI mouse model.

*Yersinia pestis* is one of the pathogens of fatal lung infections. In the pre-inflammatory phase, T3SS secretes more than a dozen effector proteins Yops, pore-forming toxins, Pla, and anti-host factors to inhibit the innate immune response, which integrally coordinates the establishment of lung infection (Rosenzweig and Chopra, 2013). This is an extensive process. There is no doubt that neutrophils are the forerunners (Banerjee et al., 2020). Pla, an adhesin produced by *Y. pestis*, inhibits IL-17 expression delaying neutrophil recruitment and degranulation functioning to silence host protective immune defense mechanisms (Theriot et al., 2023). In addition, YopM prevented inflammasome assembly (LaRock and Cookson, 2012), and YopJ inhibited cytokine production by blocking TAK1 with its downstream MAPK and NF-κB inflammatory signaling (Orning et al., 2018; Philip et al., 2014).

In the later stages of infection, there is an explosive increase in pro-inflammatory factors, which manifests as acute pulmonary congestion, edema, and necrosis in the lungs (Chung and Bliska, 2016). As research progresses, it is suggested that *Y. pestis* modulates the innate immune response by inducing cell death. Pla interferes with the Fasl-dependent exogenous apoptotic pathway, effectively suppressing the innate immune response and creating an adaptive environment for bacterial replication (Caulfield et al., 2014). Inhibition of apoptotic signaling appears to alter the direction of the innate immune response. Caspase-8 responds to *Yersinia* infection, an effect that appears to be a combined process of apoptosis, pyroptosis, and necroptosis (Orning et al., 2018; Philip et al., 2014; Malireddi et al., 2020; Zheng et al., 2021). Caspase-8 and RIPK1 are required for inflammatory cellular focalization induced by YopJ in response to TAK1 inhibition (Orning et al., 2018). Furthermore, another study added that RIPK1-dependent PANoptosis was triggered in TAK1-deficient macrophages, suggesting that *Yersinia* pre-inhibition of an effective innate immune response is followed by a positive host response that initiates caspase-8-dependent immune defense against *Yersinia* infection (Malireddi et al., 2020).

#### 7.1.3 *Pseudomonas aeruginosa* (PA)

*Pseudomonas aeruginosa* (PA) as an extracellular opportunistic pathogen, T3SS and flagellin, outer membrane vesicles, and pyocyanin are responsible for destructive immunity (Wood et al., 2023). Apoptosis tends to occur early in infection, and pusillanimins are apoptosis-related toxins that inhibit early lung inflammation favoring early bacterial multiplication and respiratory colonization (Allen et al., 2005). Outer membrane vesicles are carriers of multiple virulence factors released by PA that can detach from live bacteria to act as independent virulence factors causing lung inflammation (Park et al., 2013). Primarily contained are fat-soluble virulence factors that bind to membrane lipids to invade the cytoplasm and interfere with airway epithelial cilia to clear pathogens (Bomberger et al., 2009).

Signaling crosstalk between pyroptosis and necroptosis during PA infection is also of interest. RIPK3 kinase-dependent function drives necroptosis in epithelial cells, and NLRP3 activation is dependent on mitochondrial dysfunction and ROS production caused by the onset of necroptosis (Li et al., 2023c). The RIPK3 scaffold structural domain drives destructive lung inflammation and death, which is distinct from necroptosis triggered by the RIPK3 kinase structural domain. In a different way, blocking RIPK3 effectively ameliorates lung inflammation as well as mortality in PA infection (Lyons et al., 2023). Not only that, NLRC4 deletion in macrophages caused deficient caspase-1/3/7/8 activation, and necroptosis compensated for the lack of immune response, underscoring the occurrence of PANoptosis in macrophages in the context of PA infection (Sundaram et al., 2022).

#### 7.1.4 Other gram-negative bacilli

LPS is a virulence factor for Gram-negative bacteria (*Francisella, Klebsiella pneumoniae, Burkholderia thailandensis*) and has been strongly associated with poor sepsis outcome (Mazgaeen and Gurung, 2020). Sepsis-associated acute lung injury (SALI) is the primary cause of mortality in individuals experiencing severe sepsis. The primary mechanism of SALI involves a systemic inflammatory response induced by LPS, resulting in irreversible shock, endothelial cell injury, and ultimately lung failure (Deng et al., 2025). Mechanisms by which LPS induces SALI include synergistic action of RIPK3 with GSDMD (Chen et al., 2020), caspase-8 inhibition impedes apoptosis-dependent macrophage depletion delaying inflammatory clearance (Janssen et al., 2011), and targeting ZBP1 is critical for the regulation of PANoptosis (Guo et al., 2023; Cui et al., 2022a). Uncontrollable body inflammation disrupts normal coagulation mechanisms and systemic multi-organ functional involvement into an irreversible phase of endotoxic shock.

Caspase-11 mediates endothelial cell barrier disruption, and bacterial entry into the bloodstream, and excessive caspase-11 activation triggers shock (Cheng et al., 2017). Endotoxemia after hemorrhagic shock (HS) often promotes the irreversible development of shock, leading to multiple organ dysfunction, which can lead to fatalities (Li et al., 2018; Khazoom et al., 2020; Peitzman et al., 1995). HS induces mitochondrial and lysosomal damage and releases inflammatory mediators to destroy the homeostasis of endothelial cells and macrophages and affect the development of SALI. Two inflammatory mediators, HMGB1 and extracellular cold-inducible RNA-binding protein (eCIRP), are associated with HS and high mortality in endotoxemia (Aziz et al., 2019; Yang et al., 2016a). Initially, LPS triggers mtDNA release via the TLR4/Caspase-11/GSDMD pathway; the cyclic GMP-AMP synthase (cGSA) detects mtDNA in the cytoplasm and triggers an increase in the phosphorylation level of stimulators of the interferon genes (STING) (Platnich et al., 2018). HMGB1/RAGE induces NLRP3 activation by disrupting lysosomes to release cathepsin B (Xiang et al., 2011). Both the pro-inflammatory factors HMGB1 and CIRP can trigger ROS production and are actively involved in this process via TXNIP, a thioredoxin-interacting protein (Aziz et al., 2019; Xiang et al., 2011). Inflammation triggers CIRP from the nucleus to the cytoplasm and its release into the circulation, which can upregulate TNF-α, IL-1β, and surface adhesion molecules in the lung tissue (Yang et al., 2016b). In macrophages, eCIRP has a dual role. It induces mtDNA release through the TLR4-MyD88 pathway and promotes STING activation through the TLR4-TRIF pathway (Aziz et al., 2019; Bortolotti et al., 2018). Importantly, the detection of cytosolic DNA by the cGAS-STING axis initiates K^+^ efflux upstream of NLRP3, coordinating the lysosomal cell death program of NLRP3 (Gaidt et al., 2017; Ning et al., 2020). In addition, caspase-11 is activated early during endotoxic shock, which regulates the activity of caspase-3/7 (Kang et al., 2002). Caspase-8 and caspase-11 synergistically coordinate to drive systemic inflammation with no involvement from RIPK1 and RIPK3 (Mandal et al., 2018).

Interestingly, insufficient LPS acylation of *Francisella* is not fully recognized by caspase-11, favoring *Francisella* latency in the organism (Lagrange et al., 2018). A short immune delay is followed by a rapidly developing inflammatory response leading to severe sepsis. However, recent studies have proposed that AIM2-PANoptosis occurs to provide immunoprotection against *Francisella* infection (Wang et al., 2023). First, Pyrin recognizes *Francisella* or *herpes simplex virus-1* in response to AIM2. Deletion of the ZBP1 sensor attenuates caspase-1 processing. Pyrin and ZBP1 act synergistically on AIM2-caspase-1 activation. In response to ASC cohesion, a multiprotein complex of AIM2-PANoptosome is formed, which induces PANoptosis during infection (Lee et al., 2021).

### 7.2 Viral infection-related ALI

#### 7.2.1 SARS-CoV-2

SARS-CoV-2 is a coronavirus, and its protein components such as N proteins can effectively limit apoptosis to promote viral replication (Pan et al., 2023). Targeting apoptosis reduces disease severity, and studies have demonstrated that caspase-dependent apoptosis of airway epithelial cells occurs commonly and is less symptomatic in children and young patients compared with older adults (Inde et al., 2021).

In addition, pyroptosis can be induced by structural and nonstructural proteins (Momeni, 2011). Inflammation and thrombosis are both clinical phenomena observed during the SARS-CoV-2 infection (Zaid et al., 2020). The spike protein of SARS-CoV-2 interacts with two receptors, angiotensin-converting enzyme 2 and transmembrane serine protease 2, to facilitate virus entry into host cells (Jackson et al., 2022). Moreover, the virus can also be internalized into hematopoietic stem cells and endothelial progenitor cells, activating NLRP3 through the spike protein (Ratajczak et al., 2021). Syncytia, which is formed by the fusion of multiple cells, has been observed in deceased patients with COVID-19. *In vitro* simulations have shown that syncytia undergoes GSDME-dependent pyroptosis, which may occur in infected alveolar type II epithelial cells (Ma et al., 2021).

The role of non-structural proteins (NSP) encoded by SARS-CoV-2 in having a pyroptosis-activating effect requires investigation. There are at least 16 types of non-structural proteins, such as NSP3 and NSP4, that primarily participate in virus replication (Pahmeier et al., 2023). NSP6 targets the inactive form of ATPase H+ transporting accessory protein 1, resulting in defective lysosomal acidification and impaired autophagy. This, in turn, activates NLRP3 and mediates the pyroptosis of epithelial cells (Sun et al., 2022). Contrarily, NSP5 activates the NLRP1 inflammasome. However, in terms of the pyroptosis mechanism, NSP5 inhibits GSDMD and utilizes the caspase-3/GSDME alternative pyroptosis pathway (Planès et al., 2022).

Furthermore, during infection, vascular endothelial cell damage leads to the release of von Willebrand factor, initiating the coagulation cascade and resulting in thrombosis (Eltobgy et al., 2022). Excessive inflammation caused by bacterial or viral infection induces type I IFN, which acts as a procoagulant signal. This signal induces macrophage pyroptosis and the release of tissue factor through the caspase-11 pyroptosis pathway, mediating intravascular coagulation (Ryan et al., 2023).

Different views exist as to whether necroptosis mediates the onset of destructive inflammation during infection. ZBP1-RIPK3-MLKL is one of the important molecular mechanisms by which excessive inflammation leads to irreversible lung injury in critically ill patients (Li et al., 2023b). In contrast, *in vivo* modeling studies in SARS-Cov-2-infected mice pointed out that MLKL defects did not improve lung pathology (Stefanie et al., 2024). However, caspase-8-dependent apoptosis, necroptosis, and typical lung inflammatory features were observed in lung sections from deceased COVID-19 patients (Li et al., 2020).

The release of inflammatory mediators leads to excessive immune response, cell death, and pulmonary pathologic changes. The combination of TNF-α and IFN-γ induces nitric oxide production and drives caspase-8/FADD mediated PANoptosis through the JAK/STAT1/IRF1 axis. Cell death mediated by this mechanism tightly links cytokine storms to lung injury (Karki et al., 2021). Conversely, ZBP1 expression in immune cells was higher in deceased COVID-19 patients. ZBP1 is not only a product of viral RNA, but also a sensor for RNA or DNA viruses (Kao et al., 2014). ZBP1 promotes PANoptosis and promotes the further release of inflammatory factors (Karki et al., 2022). In conclusion, these studies suggest that inflammatory cell death is closely associated with pathological changes in COVID-19 patients.

#### 7.2.2 Influenza A virus (IAV)

In addition to coronaviruses, influenza A viruses (IAV), such as H7N9 and H9N2, induce GSDME-dependent pyroptosis in AEC (Zhang et al., 2023; Wan et al., 2022). Human bronchial epithelial cells can sense IAV dsRNA and activate GSDMD and GSDME. Inflammatory mediators produced by IAV infection are potent activators of ZBP1, and cyclic-promoting effects of necroptosis and disruptive inflammation are found in infected macrophages (Ferreira et al., 2023). However, in the absence of apoptosis, viral infection can be limited by relying independently on necroptosis (Soni et al., 2023; Shubina et al., 2020). Similar to RIPK1-PANoptosome, ZBP1-PANoptosome was discovered in response to an IAV infection. ZBP1 is not only a product of viral RNA but also a sensor of RNA or DNA viruses (Zheng and Kanneganti, 2020). ZBP1 senses Z-RNA produced by influenza viruses and interacts with NLRP3 (Zheng and Kanneganti, 2020). ZBP1-NLRP3 inflammasome is subsequently activated by recruiting RIPK3 and caspase-8; caspase-6 is required for inflammasome activation and promotes RIPK3 binding to ZBP1 (Zheng et al., 2020). Additionally, ZBP1 initiates RIPK3-MLKL, leading to DNA leakage into the cytosol, and ultimately necroptosis (Yang et al., 2020). RIPK3 can interact with RIPK1, which recruits caspase-8 through FADD to initiate apoptosis (Alvarez-Diaz et al., 2016). In response to IAV infection, these components constitute ZBP1-PANoptosom in innate immune cells, thereby protecting the host from viral infection.

### 7.3 Transfusion-related acute lung injury (TRALI)

Transfusion-related acute lung injury (TRALI) is a severe complication that can occur during or up to 6 h after a transfusion (Barrett and Kam, 2006). While the exact cause of TRALI is not fully understood, in most cases it may involve an immune-directed, antibody-mediated mechanism. This mechanism is frequently triggered by two harmful factors: the first is the patient’s underlying injury, which might include infection, sepsis, or surgery, and the second is the infusion of blood components containing antibodies or biological response modifiers (BRM), which cause neutrophils to become excited and generate TRALI symptoms (Silliman, 2006).

Interestingly, the “two-hit” model of TRALI is similar to the two-step activation of NLRP3, both of which may also be linked to each other (Land, 2013). An earlier study revealed that the development of TRALI could be uniquely reliant on polymorphonuclear leukocytes (PMNs). Human leukocyte antigen A2 promotes interaction between PMNs and endothelial cells through Src phosphorylation and increases the pro-inflammatory state through upregulation of NF-κB and NLRP3, ultimately leading to lung tissue injury by releasing more ROS (Le et al., 2022). In addition, ROS release serves as a convergence point for additional antibody-mediated TRALI-related processes (Tung et al., 2022). This could create an ideal setting for inflammasome activation. Pyroptosis-related protein expression is upregulated by ROS and can facilitate inflammasome establishment and activation in response to TXNIP (Abais et al., 2015; Zhou et al., 2010).

Nonetheless, HMGB1 is a risk factor for erythrocyte transfusion, and *in vitro* studies have shown that human lung endothelial cells undergo necroptosis upon exposure to allogeneic erythrocytes and release RIPK3 and HMGB1 (Qing et al., 2014). In the plasma of patients with severe sepsis, RIPK3 levels were increased after transfusion and were higher in non-survivors. In a mouse model, erythrocyte infusion enhanced the susceptibility of mice to LPS-induced lung inflammation by releasing HMGB1 (Ware, 2014). This necroptosis and danger signaling release may explain the mechanism underlying the increased risk of acute respiratory distress syndrome in critically ill patients receiving erythrocyte transfusions (Qing et al., 2014; Ware, 2014).

The prevention of adverse effects of hemolytic transfusion is essential for patients with sickle cell disease (SCD) who are dependent on transfusion for survival (Firth et al., 2003; Suddock and Crookston, 2024). SCD is characterized by chronic hemolytic anemia. Recent studies confirm that NLRC5 interacts with NLRP12-PANoptosome to drive inflammatory cell death in hemolytic models (Sundaram et al., 2024b). Repeated hemolysis in the blood vessels of SCD leads to endothelial dysfunction and ischemia-reperfusion injury (I/RI). SCD-induced I/RI drives organ damage, with the lung being the most vulnerable organ (Ansari and Gavins, 2019). In the context of lung ischemia-reperfusion injury (LI/RI), it is noteworthy that the activation of NF-κB/NLRP3 occurs due to the release of IL-1R by monocytes, resulting in damage to the endothelium cells (Zhou et al., 2021). Pathological tests have demonstrated that renal or intestinal ischemia-reperfusion injury (RI/RI or II/RI) can induce ALI, which is marked by notable inflammation and lung damage (Liu et al., 2020b; Li et al., 2022). Apoptotic signals govern pyroptosis during the investigation of the intestinal ischemia-reperfusion injury. PKR-like ER kinase, a protein found in mitochondria-associated membranes, initiates apoptotic signals. Furthermore, MAMs recruit NLRP3 and facilitate its assembly and activation in response to stimuli. Suppression of PERK can also hinder pyroptosis signaling and mitigate II/RI (Li et al., 2023d). Nrf2 regulates heme-oxygenase 1, a route that safeguards the body against detrimental stimuli and can ameliorate RI/RI (Li et al., 2023e). Similarly, under the influence of recombinant HMGB1, this pathway is stimulated and improves LI/RI (Fei et al., 2020).

### 7.4 Ventilation-related ALI (VILI)

Mechanical ventilation (MV) is often operated to maintain respiratory function in critically ill patients such as preterm infants with respiratory distress and hemorrhagic shock (Davis et al., 2024). However prolonged high tidal volume MV increases the risk of bronchopulmonary dysplasia in preterm infants. Lung hyperexpansion and hyperoxia are key factors in VILI (van Kaam, 2024).

At the cellular level, VILI is accompanied by various forms of cell death (Kuipers et al., 2012; Shao et al., 2022). Activation of the NLRP3 inflammasome was found to be necessary in a double-hit VILI study model by LPS and MV. Caspase-1 and NLRP3 were expressed in the alveolar lavage fluid of patients with MV, and inhibiting IL-1β could alleviate hypoxemia (Kuipers et al., 2012; Kuipers et al., 2011). Plasma RIPK3 levels were higher in patients with MV compared to those without MV, and mouse RIPK3 deficiency ameliorated VILI (Shao et al., 2022). Patients with ALI are often exposed to hyperoxia before the application of MV. After exposure to hyperoxia, HV activates caspase-8/9, indicating that death receptors or mitochondria mediate the occurrence of endogenous and exogenous apoptosis, causing severe lung injury (Makena et al., 2010). Importantly hyperoxia drives ROS production directly or indirectly involved in cell death (Alva et al., 2023). In many diseases, ROS levels are positively correlated with PANoptosis occurrence. For example, targeting ROS production can alleviate organ damage associated with PANoptosis occurrence in inflammatory diseases such as sepsis (Ding et al., 2024). Existing studies suggest that systemic high inflammation levels as well as extrapulmonary organ damage are observed in the VILI mouse model. For example, the pro-inflammatory mediator HMGB1 promotes hepatic PANoptosis (Ding et al., 2023).

## 8 Therapeutic agents targeting apoptosis, pyroptosis, necroptosis, and PANoptosis in ALI

### 8.1 Caspase family inhibitors

Caspase family proteins, which are involved in apoptosis, pyroptosis, PANoptosis, and regulation of necroptosis, were targeted for inhibition to alleviate the severity of ALI.

Inhibitors of caspase-1 include Ac-YVAD-CMK, VX-765, and Ac-YVAD-CHO.The mechanism of action of caspase-1 inhibition differs based on the structural specificity of the inhibitors involved (Kasana et al., 2024). Ac-YVAD-CMK is an irreversible and highly selective inhibitor of caspase-1 (Mathiak et al., 2000). Ac-YVAD-CMK exhibits greater selectivity for caspase-1 compared to the broad-spectrum caspase inhibitor Z-VAD-FMK and does not disrupt apoptosis-related pathways, thereby minimizing off-target effects (Modi et al., 2023). In contrast to the irreversible inhibitor Ac-YVAD-CMK, Ac-YVAD-CHO interacts dynamically with the target through the aldehyde group, resulting in a reduction of the inhibitory effect as concentration decreases (Garcia-Calvo et al., 1998). VX-765, an oral reversible caspase-1 inhibitor entering clinical trials for inflammatory diseases (Modi et al., 2023), met all pharmacokinetic and pharmacodynamic targets in Phase I. In 2005, 68 psoriasis patients participated in a 4-week Phase IIa safety and pharmacokinetic study (Kasana et al., 2024). To date, there have been no new developments in clinical trials involving VX-765.

Experimental modeling studies at ALI have found, that Ac-YVAD-CMK and VX-765, potent inhibitors of caspase-1, were effective in protecting against alveolar macrophage and pulmonary vascular endothelial cells brought on by SARS-CoV-2 infection and LI/RI injury (Pan et al., 2021; Wu et al., 2015; Wu et al., 2021). In addition, for another caspase-1 inhibitor, Ac-YVAD-CHO, it was proposed that nebulized inhalation was effective in alleviating LPS-induced endotoxemia in rats, and this mode of administration is more suitable to be applied to the clinical administration of ARDS (Boost et al., 2007). Moreover, caspase-3 and caspase-8 inhibitors are also targets for alleviating sepsis and endotoxic shock. Z-DEVD-FMK targets caspase-3 and blocks caspase-3/GSDMD pyroptosis signaling (Qin et al., 2024). Z-IETD-FMK inhibits caspase-8 and induces neutrophil-resident clearance of bacteria to eliminate lung inflammation (Lentini et al., 2023). Importantly, Ac-FLTD-CMK, a caspase-1/4/5/11 specific inhibitor, specifically inhibits FLTD peptide, the cleavage site of inflammatory caspases binding to GSDMD in macrophages (Yang et al., 2018). Inhibition of apoptotic caspases has emerged as a therapeutic target for IAV infection. Q-VD-OPh, which broadly inhibits a wide range of apoptotic caspases, blocks IAV replication in bronchial epithelial cells (Soni et al., 2023). The combined inhibition of apoptotic and inflammatory caspases provides a more comprehensive approach to disease treatment. Z-VAD-FMK, which broadly inhibits caspases, effectively ameliorates LI/RI, inhibits LPS-mediated apoptosis to enhance mouse survival and ameliorates endothelial damage associated with PA infection (Wang et al., 2020c; Kawasaki et al., 2000; Le Berre et al., 2004). In conclusion, targeted inhibition of the caspase family has become an important target for ALI therapy (Table 2).

**TABLE 2:** Caspase family inhibitors in ALI models.

Name	Target	Effects	Experimental models	Ref
Ac-YVAD-CMK	Caspase-1	Targeted inhibition of caspase-1	LPS-induced ALI model in mice, SARS-CoV-2-induced ALI model in mice	Pan et al. (2021), Wu et al. (2015)
VX-765	Caspase-1	Targeted inhibition of caspase-1	LI/RI mouse model	Wu et al. (2021)
Ac-YVAD-CHO	Caspase-1	Targeted inhibition of caspase-1	LPS-induced endotoxemia model in rats	Boost et al. (2007)
Z-DEVD-FMK	Caspase-3	Specific inhibition of caspase-3	Cecal ligation to model sepsis-induced lung injury in mice	Qin et al. (2024)
Z-IETD-FMK	Caspase-8	Selective inhibition of caspase-8	A mouse model of lethal bacterial peritonitis and pneumonia	Lentini et al. (2023)
Wedelolactone	Caspase-11	Inhibition of NF-κB signaling-mediated	Bacterial LPS-stimulated mouse BALB/c 3T3 cell model	Kobori et al. (2004)
Goitrin	Caspase-11	Inhibition of caspase-11	LPS-induced septic shock mouse model	Ruan et al. (2023)
Ac-FLTD-CMK	Caspase-1/4/5/11	Specific inhibition of inflammatory caspases binding to GSDMD at the cleavage site FLTD peptide	LPS-stimulated RAW264.7 cells	Yang et al. (2018)
Q-VD-OPh	Caspase-1/3/7/8/9/10/12	Widespread inhibition of caspase-1/3/7/8/9/10/12	Mouse model of IAV-induced lung inflammation	Soni et al. (2023)
Z-VAD-FMK	Caspase-1/3/4/5/7/8/11	Widespread inhibition of caspase-1/3/4/5/7/8/11	Rat model of LI/RI, LPS-induced ALI model in mice, PA-infected rat lung injury model	Wang et al. (2020c), Kawasaki et al. (2000), Le Berre et al. (2004)

### 8.2 Inflammasomes inhibitors (NLRP1/3, NLRC4, AIM2)

The inflammasome is a core member of pyroptosis and an important component of the PANoptosome. MCC950, a small molecule inhibitor of NLRP3 pharmacology, selectively blocks the interaction of NLRP3 with NEK7 and has very significant therapeutic effects in a wide range of inflammatory diseases (Pan et al., 2021). Examples include SARS-CoV-2 and LPS-induced lung infections, acute kidney injury, colitis, and myocardial injury (Pan et al., 2021; Wang et al., 2021).

Britannin and alantolactone, two bioactive natural compounds from traditional Chinese medicine, demonstrate significant inhibitory effects on NLRP3 inflammasome activation via distinct mechanisms (Shao et al., 2024). Both drugs directly target the NACHT domain of NLRP3, which disrupts its oligomerization and subsequent assembly with NEK7, according to structural and functional assessments (Li et al., 2023f). Their inhibitory effects function independently of NLRP3 ATPase activity, setting them apart from traditional ATP-competitive inhibitors like MCC950 (Wang et al., 2021).

The interaction of ADS032 with the NACHT domains of NLRP1 and NLRP3 identifies it as the inaugural dual-specific inhibitor for these inflammasomes. Additionally, it is the first NLRP1-selective compound to exhibit cross-species efficacy in both murine and human cellular models. This property addresses a significant translational barrier resulting from interspecies differences in NLRP1 activation mechanisms (Docherty et al., 2023). In a murine IAV infection model, ADS032 demonstrated broad therapeutic effects over time, protecting during both the early phase of viral replication and the late phase characterized by an inflammatory cytokine storm. MCC950 exhibited time-dependent variations in efficacy; it was effective when administered early, but its delayed application paradoxically heightened susceptibility to low-dose IAV challenge (Tate et al., 2016).

JC2-11, a derivative of benzylideneacetophenone synthesized from the chalcone scaffold, exhibits broad-spectrum inhibitory activity against various inflammasomes, including NLRP3, NLRC4, and AIM2 (Lee et al., 2022). The pan-inflammasome suppression strategy effectively bypasses compensatory activation mechanisms, which is a significant limitation of single-target inhibitors (Heinisch et al., 2022).

Both ADS032 and JC2-11 present innovative strategies for inflammasome modulation; however, their potential for translation into clinical applications has not been thoroughly investigated. Current limitations encompass inadequate pharmacokinetic profiling in non-rodent species and unvalidated efficacy in complex disease models that exhibit interactions between canonical and non-canonical inflammasome pathways (Docherty et al., 2023; Lee et al., 2022). Systematic structure-activity relationship studies and multi-omics validation are crucial for addressing these gaps and advancing these compounds toward clinical development (Table 3).

**TABLE 3:** Inflammasome inhibitors in ALI models.

Name	Target	Effects	Experimental models	Ref
ADS032	NLRP1/NLRP3	Dual inhibition of NLRP1 and NLRP3	IAV-induced lung inflammation model in mice	Docherty et al. (2023)
Britannin	NLRP3	Blocking the interaction between NLRP3 and NEK7	LPS-induced ALI mouse model	Shao et al. (2024)
Alantolactone	NLRP3	Binds to the NACHT structural domain of NLRP3 to inhibit the activation and assembly of NLRP3 inflammatory vesicles	LPS-induced ALI mouse model	Li et al. (2023f)
MCC950	NLRP3	Selective inhibition of NLRP3	SARS-CoV-2-induced ALI mouse model, LPS-induced ALI mouse model	Pan et al. (2021), Wang et al. (2021)
JC2-11	NLRP3/NLRC4/AIM2	Pan-inflammasome inhibitors	LPS-induced ALI mouse model	Lee et al. (2022)

### 8.3 RIPK1/RIPK3 inhibitors

The RIPK1 inhibitors that have been developed can be categorized into three types based on how they bind to RIPK1, type I and type II are ATP-competitive and bind to the ATP-binding site, and type III binds to the allosteric pocket around the ATP-binding site (Shi et al., 2022). Necrostatin-1 and GSK2982772 are both type III RIPK1 inhibitors and GSK2982772 is in phase II clinical trials (Shi et al., 2022). Necrostatin-1 is widely used to inhibit necroptosis in several animal models of inflammation, including LPS and mechanical ventilation-induced ALI, and TNF-induced systemic inflammatory response syndrome (SIRS) (Takahashi et al., 2012; Lin et al., 2020). Necrostatin derivatives, such as Necrostatin −2 and Necrostatin −5, alleviate the harmful inflammation caused by bacteria, SARS-CoV-2, and I/RI (Wang et al., 2007; Hao et al., 2023; Guo et al., 2024).

ZB-R-55 signifies a significant advancement in the development of RIPK1 inhibitors due to its innovative dual-targeting mechanism, which simultaneously interacts with both the allosteric regulatory pocket and the ATP-binding catalytic domain of RIPK1(Yang et al., 2022). This bispecific interaction strategy addresses the limitations of traditional single-mode inhibitors by synergistically stabilizing inactive kinase conformations and competitively inhibiting ATP hydrolysis. In comparison to GSK2982772, a first-generation allosteric RIPK1 inhibitor in Phase II clinical trials, ZB-R-55 demonstrates significantly greater inhibitory potency, improved kinase family selectivity, and optimized oral pharmacokinetic properties (Shi et al., 2022; Yang et al., 2022).

GSK872 is a classic RIPK3-selective inhibitor with favorable therapeutic effects in ALI, Alzheimer’s disease, acute kidney injury, spinal cord injury, and systemic inflammatory diseases (Cui et al., 2022b; Zhong et al., 2023).

In addition, UH15-38 addressed the hyperinflammatory state caused by IAV infection without affecting adaptive immunity (Gautam et al., 2024).

Unlike the classical inhibitor GSK872, UH15-38 utilizes a distinct binding mode characterized by significant interactions with both the hinge region and the back pocket of the RIPK3 active site, thereby improving its inhibitory potency and intracellular efficacy (Gautam et al., 2024). The therapeutic potential has been validated in IAV infection models, indicating promising prospects for clinical translation in necroptosis-related diseases (Willson, 2024).

HG-9-91-01, a salt-inducible kinase inhibitor, inhibits TNF/TLRs-mediated necroptosis by promoting the interaction between RIPK1 and RIPK3 while decreasing the binding of RIPK3 to MLKL and the oligomerization of MLKL. The multimodal mechanism inhibits necroptosis while simultaneously activating apoptosis and pyroptosis via cross-regulation of signaling pathways, illustrating its polypharmacological bioactivity and therapeutic potential for inflammatory diseases (Huang et al., 2022). In conclusion, RIPK1/RIPK3 are involved in PANoptosome composition and are important targets for disease intervention (Lee et al., 2022) (Table 4).

**TABLE 4:** RIPK1/RIPK3 inhibitors in ALI models.

Name	Target	Effects	Experimental models	Ref
Necrostatin-1	RIPK1	Type Ⅲ RIPK1 inhibitor	Ventilator-induced ALI mouse model, TNF-induced SIRS mouse model, LPS-induced ALI model in mice	Takahashi et al. (2012), Lin et al. (2020), Wang et al. (2007)
Necrostatin-2	RIPK1	Pharmacological inhibitor of RIPK1	SARS-CoV-2 stimulates human airway epithelial cells	Hao et al. (2023), Guo et al. (2024)
Necrostatin-5	RIPK1	Non-pharmacological inhibitor of RIPK1 Necrostatin Derivatives	Mouse model of bacterial pneumonia	González-Juarbe et al. (2015), Wang et al. (2007)
GSK2982772	RIPK1	Type Ⅲ RIPK1 inhibitor	Clinical trial phase (psoriasis, rheumatoid arthritis, and ulcerative colitis)	Shi et al. (2022)
ZB-R-55	RIPK1	Dual-mode RIPK1 inhibitor	LPS-induced SIRS and sepsis mouse model	Yang et al. (2022)
GSK872	RIPK3	Binding of the RIPK3 kinase structural domain	LPS-induced ALI mouse model	Cui et al. (2022b), Zhong et al. (2023)
UH15-38	RIPK3	Targeted inhibition of RIPK3	IAV-induced severe lung injury model in mice	Gautam et al. (2024)
HG-9-91-01	RIPK3	Inhibition of RIPK3 kinase activity	TNF-induced SIRS mouse model	Huang et al. (2022)

### 8.4 GSDMD/GSDME/MLKL inhibitors

GSDMD/GSDME/MLKL are the ultimate determinants of PANoptosis occurrence. Ac-FLTD-CMK specifically inhibits inflammatory caspases, which is equivalent to being a derivative inhibitor of GSDMD and can control macrophage pyroptosis *in vitro* (Yang et al., 2018). Necrosulfonamide (NSA), Disulfiram, and Dimethyl fumarate (DMF) are known GSDMD inhibitors (Rathkey et al., 2018; Adrover et al., 2022; Humphries et al., 2020). Among them, NAS can inhibit MLKL by binding to Cys86 of MLKL (Hao et al., 2023).

NSA uses a “single agent, dual mechanisms” approach to inhibit GSDMD and MLKL, breaking their synergistic interaction and reducing inflammatory amplification cascades (Hao et al., 2023; Rathkey et al., 2018). Disulfiram, an FDA-approved anti-alcohol, and strong GSDMD inhibitor, reduces mouse pyroptosis and LPS-induced septic mortality (Hu et al., 2020). New research suggests it protects against TRALI models and SARS-CoV-2 infection by inhibiting neutrophil extracellular traps (NETs) formation (Zhao et al., 2023). Reducing pulmonary NETs, neutrophil infiltration, and vascular leakage reduces lung histological damage, suggesting repurposing for inflammatory pulmonary diseases (Adrover et al., 2022). Through succinylation-mediated functional regulation of GSDMD, DMF, an FDA-approved treatment for multiple sclerosis and associated conditions, reduces inflammation (Humphries et al., 2020). This novel post-translational modification mechanism provides molecular-level validation for the therapeutic efficacy of DMF in inflammatory disease (Bresciani et al., 2023). GW806742X can also target the inhibition of MLKL (González-Juarbe et al., 2015). In addition, it should be added that Ac-DMLD-CMK targets inhibition of caspase-3/GSDME signaling to alleviate sepsis-induced lung injury (Qin et al., 2024). We summarize the inhibition targets and the therapeutic effects exerted in different ALI models in the table (Table 5).

**TABLE 5:** GSDMD/GSDME/MLKL inhibitors in ALI models.

Name	Target	Effects	Experimental model	Ref
Ac-FLTD-CMK	GSDMD	Specific inhibition of inflammatory caspases binding to GSDMD at the cleavage site FLTD peptide	LPS-stimulated RAW264.7 cells	Yang et al. (2018)
NSA	GSDMD	Binding to GSDMD Cys191 inhibits GSDMD	LPS-induced mouse sepsis model	Rathkey et al. (2018)
Disulfiram	GSDMD	Inhibition of GSDMD by binding Cys191 and NETs	LPS-induced mouse model, LPS and anti-H2d antibody-induced TRAIL mouse model, SARS-CoV-2-induced ALI mouse model	Adrover et al. (2022), Hu et al. (2020), Zhao et al. (2023)
DMF	GSDMD	Succinates GSDMD and prevents binding to caspase-1	LPS-induced shock mouse model	Humphries et al. (2020)
Ac-DMLD-CMK	GSDME	A peptide targeting caspase-3/GSDMD signaling	Cecal ligation to model sepsis-induced lung injury in mice	Qin et al. (2024)
GW806742X	MLKL	Targeted inhibition of MLKL	Mouse model of bacterial pneumonia	González-Juarbe et al. (2015)
NSA	MLKL	Inhibition of MLKL by binding to Cys86 of MLKL	SARS-CoV-2 stimulates human airway epithelial cells	Hao et al. (2023)

### 8.5 Calpain inhibitors

The regulation of apoptosis, pyroptosis, necroptosis, and PANoptosis by caspases has been widely studied (Pandeya and Kanneganti, 2024). During our literature survey, we identified another protein family member that also seems to be involved in caspase regulation as well as cell death. Calpains figure among apoptosis (Momeni, 2011), pyroptosis, and necroptosis (Davis et al., 2019; Cheng et al., 2018). Consider whether inhibiting it could also impede PANoptosis and treat ALI and other inflammatory illnesses. More research is needed to prove this conjecture.

Calpain-1 and -2 are the best-researched calpains and are expressed in mammalian tissues (Wang et al., 2020d). Calpain inhibitors diminish neuronal death and limit disease progression (Ono et al., 2016). Calpain inhibitors such as PD150606, SNJ-1945, and MDL28170 are highly selective for calpain and have inhibitory effects on both calpain-1 and calpain-2 (Donkor, 2020; Dókus et al., 2020). PD150606 inhibits calpain activity by binding to the CysPc structural domain of calpain, thereby attenuating neuroinflammation and apoptosis (Wang et al., 1996). SNJ-1945 reduced neuroinflammation, demyelinating lesions, T-cell infiltration, and neuronal damage in the multiple sclerosis model by inhibiting microglia activation and inflammatory factor production (Trager et al., 2014). Calpain inhibitors may treat other disorders. In myocardial ischemia-reperfusion injury, MDL28170 prevents cardiomyocyte apoptosis, lowers myocardial injury, and maintains mitochondrial function via inhibiting mitochondrial permeability transition pore opening (Thompson et al., 2016).

Calpain hydrolyzes vascular endothelial cadherin in lung microvascular cells, leading to the disruption of the endothelial barrier and the development of pulmonary edema. Additionally, serum calpain levels may serve as a potential biomarker for ALI, which is significant for assessing the severity of lung injury and predicting clinical outcomes (Yi et al., 2022; Song et al., 2023). The administration of MDL28170 to inhibit calpain activity significantly decreased pulmonary edema and alveolar wall thickening, suggesting that calpain is involved in LPS-induced endothelial barrier dysfunction in the pulmonary vasculature during ALI (Song et al., 2023). The involvement of calpains in the regulation of apoptosis, pyroptosis, and necroptosis has been addressed; however, the potential impact of calpain inhibitors on caspase-mediated cell death in ALI remains underexplored. Further discussion and investigation into this area would be beneficial.

## 9 Conclusion

Caspase-mediated PCDis common in bacterial and viral infections, blood transfusion, and ventilator-inducedALI. Initially, the various programmed death mechanisms were considered to be mutually independent, however, this perspective was challenged by the investigation of PANoptosis, which involves TNF-α and IFN-γ, as well as ZBP1, RIPK1, NLRC4, NLRC5, NLRP12, and AIM2 pathways. We found that the signaling crosstalk of apoptosis, pyroptosis, and necroptosis mediated PANoptosis. Although the three programmed deaths are not comprehensively involved in all lung diseases, there is evidence of the presence of partial caspase signaling crosstalk. The existence of ALIinduced by various causes poses a major threat to human health. Due to the complexity of the mechanisms involved in PCD, therapeutic agents targeting this process remain under development. Numerous studies have investigated various mechanisms of PCD and the impacts of targeted inhibition. This summary outlines the therapeutic mechanisms and effects of five classes of inhibitors: the caspase family, inflammasomes, RIPK1/3, GSDMD/GSMDE/MLKL and calpains in experimental models. Single-targeted inhibitors appear insufficient to fully ameliorate the ALI condition, potentially due to the activation of compensatory mechanisms related to cell death. Different components of SARS-CoV-2 activate NLRP3 and NLRP1 independently, indicating that a single NLRP3 inhibitor cannot inhibit NLRP1. In contrast, the dual NLRP1 and NLRP3 inhibitor ADS032 demonstrates a significant effect solely in the treatment of IAV infection. The development and application of single-agent multi-target inhibitors is forthcoming. The potential role of calpain inhibitors in mitigating caspase-mediated cell death is acknowledged; however, the mechanism by which they regulate PANoptosis remains unclear. The potential effects of calpain inhibitors on caspase-mediated cell death in ALI are not yet fully investigated. Additional exploration and investigation in this domain would be advantageous. Nonetheless, these inhibitors encounter numerous challenges during clinical translation, including target specificity, pharmacokinetic properties, safety, and potential side effects associated with long-term use. Addressing these issues necessitates comprehensive basic research and extensive clinical trials to facilitate the effective transition of these inhibitors from laboratory settings to clinical applications.
